# Unicompartmental knee arthroplasty, an enigma, and the ten enigmas of medial UKA

**DOI:** 10.1186/s10195-020-00551-x

**Published:** 2020-09-02

**Authors:** Anurag Mittal, Prashant Meshram, Woo Hyun Kim, Tae Kyun Kim

**Affiliations:** 1 TK Orthopedic Surgery, 55 Dongpangyo-ro, Bundang-gu, Seongnam-si, Gyeonggi-do 13535 Republic of Korea; 2 Department of Orthopaedics, Johns Hopkins Medical Institute, 2360 West Joppa Road, Suite 306, Baltimore, MD 21093 USA

**Keywords:** Unicompartmental knee arthroplasty, UKA, Cementless, Cost effectiveness, Indications, Bearing surface

## Abstract

Unicompartmental knee arthroplasty (UKA) is a bone- and ligament-sparing alternative to total knee arthroplasty in the patients with end-stage single-compartment degeneration of the knee. Despite being a successful procedure, the multiple advantages of UKA do not correlate with its usage, most likely due to the concerns regarding prosthesis survivability, patient selection, ideal bearing design, and judicious use of advanced technology among many others. Therefore, the purpose of this study is to review and summarize the debated literature and discuss the controversies as “Ten Enigmas of UKA.”

## Introduction

After the discovery of Ahlback in 1968 [1] that 85% of knees with clinical osteoarthritis (OA) have isolated medial compartment degeneration, modern modular unicompartmental knee arthroplasty (UKA) was conceptualized, thereby revolutionizing the knee arthroplasty. Since then, UKA has been an effective and minimally invasive alternative to total knee arthroplasty (TKA) that selectively replaces the damaged compartment of the knee with end-stage disease.

Historically, in 1954, McIntosh and Hunter [2] performed the first unicompartmental interpositional arthroplasty, followed by McKeever’s [3] attempt of using tibial plateau prosthesis in the 1960s. In the early 1970s, St. Georg Sled [4], which was the first modular UKA, was developed by Bucholz, and ever since, the prosthetic design and its kinematics have undergone refinements with the aim of providing better clinical outcomes. Oxford knee introduction by Goodfellow and Connor [5] in the 1980s heralded a new era in UKA and has been the biggest advancement in the field of partial knee arthroplasty.

**Fig. 1:**
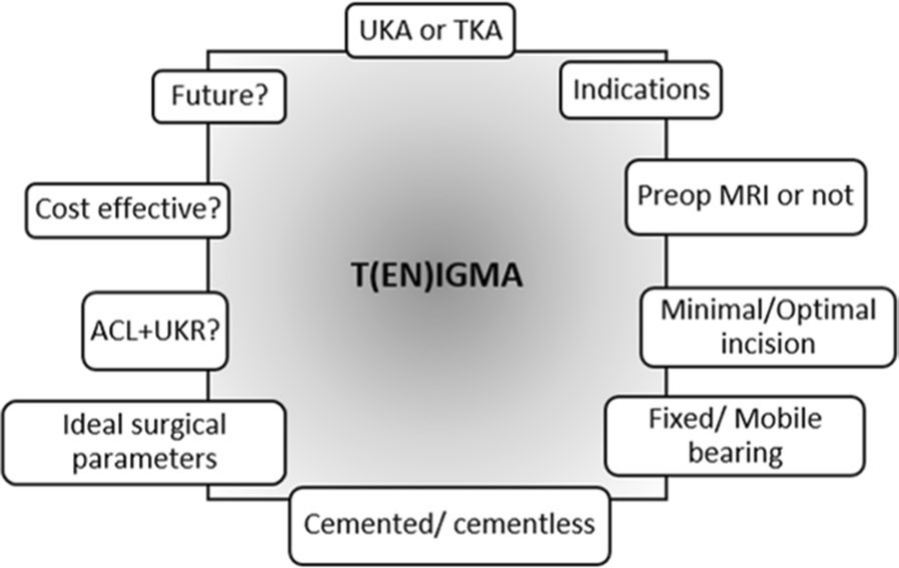
Ten Enigmas (TENIGMAs) of UKA

Despite the newer advancements over past decades and proven advantages of minimally invasive UKA, surgeons are still reluctant to use this procedure in spite of its indication. This is most likely due to the concerns regarding survivability, patient selection, ideal bearing design, and judicious use of advanced technology among many others. The purpose of this study was to summarize the controversies pertaining to UKA as “Ten Enigmas of UKA” and review the relevant literature to highlight the available evidence related to these enigmas (Fig. 1).

1. If you had a dilemma in using UKA or TKA in an UKA-indicated patient, would you choose UKA?
2. Are classical ideal and nonideal indications of UKA proposed by Kozinn and Scott still valid with newer advanced prostheses?
3. Preoperative MRI: Do they have a role in the decision-making while considering UKA in a patient?
4. Errors in component placement: Should incision be minimal or optimal?
5. Choice of bearing design: Mobile or fixed bearing?
6. Should we use cementless implants instead of cemented ones?
7. Ideal limb alignment and optimal position of UKA prosthesis: What is the consensus?
8. Anterior cruciate ligament (ACL) reconstruction simultaneously with UKA: Is it too much?
9. Is UKA a cost-effective surgery?
10. Robotics, computer navigation, and patient-specific instrumentation and implants: Conventional versus technology-assisted UKA?

### If you had a dilemma in using UKA or TKA in an UKA-indicated patient, would you choose UKA?

There is a huge burden of knee osteoarthritis in healthcare and the trends suggest that the number will continue to rise substantially due to the aging population and increased prevalence of risk factors, in particular, obesity. Subsequently, the demand for knee arthroplasty is expected to grow by more than 600% by 2030 [6]; and thus, it becomes important that patients are offered a safe and effective treatment.

UKA is considered to be a highly effective treatment after failure of conservative or joint-preserving methods for isolated osteoarthritis in the medial compartment and offers some essential short-term and long-term advantages compared with TKA. UKA has shorter operative time, reduced hospital stays, lower blood loss (reducing the number of transfusions), greater postoperative range of motion, and a higher level of activity at the time of hospital discharge [7]. Long-term benefits include preservation of bone stock for revision surgery, shorter and early recovery, lower morbidity, higher functional activity due to normal knee kinematics, and a subjective feeling of the normal knee due to preservation of the anterior and posterior cruciate ligaments and a part of the meniscus [8]. Due to extensive exposure required to perform TKA, Weale et al. observed that patella baja is more common in TKA but is rather a rare entity in UKA, which may, in part, explain the better functional results after UKA [9]. The early postoperative complications such as myocardial infarction, venous thromboembolism, cerebrovascular events, and deep infection are seen in fewer patients undergoing UKA than those undergoing TKA. Zuiderbaan et al. showed far better joint forgettability scores in UKA when compared with TKA patients [10]. Six months after surgery, Friesenbichler et al. observed that UKA patients showed better short-term quadriceps strength and gait function compared with TKA patients, together with less self-reported knee pain and stiffness [11]. Moreover, several trials report a high percentage, up to 20%, of unsure or dissatisfied patients after TKA, most of them with seemingly well-fixed and well-positioned components [12].

Despite being a less invasive and having aforementioned advantages to TKA, surgeons are reluctant to consider UKA in the indicated cases. There seem to be two main reasons for the under-use of UKA. First being the reporting of a high revision rate of UKA in national joint registries (NJRs) data [13] and second being the long learning curve of UKA compared with TKA [14]. For instance, the revision rate is 3.2 times higher for UKA than TKA in the NJR for England, Wales, and Northern Ireland [15], the largest joint registry in the world. Literature indicates that around 47% of the knee osteoarthritis patients are eligible for UKA [16], however, UKA usage is a mere 5–8% [17, 18]. The high revision rate of UKA in NJR is in contrast with the cohort studies from high-volume centers that report similar or even better survivability of the UKA compared with TKA [19]. Also, the UKA revision rate is reported to be four times higher by lowest-volume surgeons when compared with highest-volume surgeons [20]. Thus, we envisage to comprehend the causes behind this difference and also understand the probable reasons for UKA high revision rate.

*UKA is more easily revised*: Knee arthroplasty surgeons are more comfortable in revising UKA than TKA because revision of an UKA tends to be less technically challenging. Evidence suggests that more tibial stems, metal augments, and stabilized components are required when a TKA is revised in comparison with a UKA [21]. Thus, the higher revision rate of UKA should not be considered a problem because it is a manifestation of an advantage of easy revision.

*Lower threshold for revision of UKA than TKA*: Surgeons are more likely to revise UKA than TKA even with similar Oxford knee scores (OKS). Among patients with OKS < 20 (indicating poor outcome), only 12% of TKAs were revised in contrast to 63% of UKAs with the same score [22]. So, the higher revision rate does not suggest that UKA has a worse outcome than TKA.

*Selection bias*: Patients selected for UKA by surgeons tend to be younger and more active, as they are the ones who meet the ideal indications for UKA surgery. Surgeons are worried about the progression of arthritis in the opposite compartment which is one of the main reasons for revision of an UKA. Patients with a pristine lateral and patellofemoral compartment with medial compartment arthritis are selected for UKA. However, this creates a pattern of high revision rates seen with UKA [23], younger patients naturally live longer than elderly patients, thus increasing the likelihood of future revision. Also, it has been shown that the results of arthroplasty in early arthritis lead to suboptimal results compared with arthroplasty in end-stage degeneration [19, 24], which can again lead to early revision. Thus, the tendency to select younger patients for UKA with less advanced osteoarthritis could lead to higher revision rate.

*Lower threshold of error in implant positioning*: Given that there is a longer learning curve for UKA, errors are more probable to happen in component positioning compared with TKA. Additionally, these errors are less tolerated in UKA in comparison with TKA which is somewhat forgiving in terms of alignment and soft-tissue balancing. Over- or under-correction of leg alignment and the tibial component malpositioning in small magnitudes are associated with an increased risk of failure. The changes from the native joint line of more than just 3° in the coronal plane and 2° in the sagittal plane were associated with decreased prosthesis survival [14, 25]. Thus, even if a similar error occurs while performing UKA and TKA, the UKA patient might be more likely to have an early revision.

*An additional mode of failure in UKA*: Apart from all the causes of revision that are similar to those of TKA, such as aseptic loosening, polyethylene wear, and tibial subsidence, there is an additional mode of failure in UKA: the progression of arthritis in unreplaced compartments [26].

*Variability in UKA usage among surgeons* UKA usage is defined as the proportion of patients requiring arthroplasty of the knee who are offered UKA [20]. There is a wide variation of UKA usage among surgeons, ranging from 0 to 50%. Surgeons interpret the registry data and literature review about relative benefits and risks of UKA and TKA in different ways. Optimal outcomes are seen when UKA usage is between 40% and 60%, whereas acceptable revision rate is achieved with a UKA usage > 20%, and with a  < 5% usage, there is more probability of a high revision rate for that surgeon as per the study by Liddle et al. [27]. They concluded that the proportion of UKA performed is more important than the absolute number of arthroplasties performed per year. Similarly, Hamilton et al. performed a metaanalysis on the association of caseload and usage in determining outcomes of UKA and found that usage is more important than caseload [16]. The probable reason for superior functional outcomes with increased UKA usage is the improvement in the technical and nontechnical skills (proper selection of patients and preoperative workup) of the surgeon.

As shown above, several biases and reasons lead to a perceived high revision rate of UKA. In multiple comparative studies, the Oxford knee score (OKS), the Knee Society score (KSS), and the Western Ontario and McMaster Universities Osteoarthritis index (WOMAC) indicating functional outcomes were higher following UKA than TKA [28–37] (Table 1). Thus, UKA, when indicated, could be viewed as a definitive treatment option as opposed to a first stage surgery before TKA. One of the ways to optimize clinical outcomes after UKA is to increase its usage to more than 20% for optimal results as shown by Liddle et al. [27]. This can be done by widening its indications, which brings us to our next controversy.

**Table 1:** Summary of clinical outcomes and survivorship in studies comparing UKA and TKA

S. no.	Author	Year	Type of study	Age, mean (years)		Number of patients		Follow-up, mean (years)		Prosthesis implanted		Functional scores, mean		Range of motion, mean (°)		Survivorship (%)	
				UKA	TKA	UKA	TKA	UKA	TKA	UKA	TKA	UKA	TKA	UKA	TKA	UKA	TKA
1	Siman et al. [28]	2017	Retrospective	80.1	79.6	120	188	3.5	4.6	Multiple	Multiple	KSS (F) 85.4	84	119.3	111.3	98.3	98.8
2	Lum et al. [29]	2016	Retrospective	63.3	65.7	201	189	5.4	5.5	Oxford	Vanguard Zimmer	KSS (K/F)78/77.6	66/66	118.7	111.6	98.5	96.8
3	Horikawa et al. [30]	2015	Retrospective	74	72.2	28	50	9	10.5	Stryker EIUS	Stryker Scorpio NRG	JOA 80.2	81.1	142.5	126	84	92
4	Bolognesi et al. [31]	2013	Retrospective	74.2	74.6	2844	64,932	—	—	—	—	—	—	—	—	82	96.3
5	Lyons et al. [32]	2011	Retrospective	66.02	67.65	279	5606	7.12	6.5	Multiple	Multiple	KSS (K/F) 79.55/65.74	90.58/89.72	—	—	90	95
6	Costa et al. [33]	2011	Prospective	73	73	34	34	5	5	EIUS Zimmer	Stryker Scorpio NRG	KSS (K/F) 96/91	96/91	—	—	85	100
7	Sun et al. [34]	2010	Prospective	60	61	28	28	~ 4	~ 4	Oxford	AGC Biomet	KSS (F) 80.5	78.9	117	115	75	100
8	Newman et al. [35]	2009	Prospective	69.6	69.8	52	50	15	15	St. Georg Sled	Kinematic modular	BKS 92	87.5	—	—	89.8	78.7
9	Amin et al. [36]	2006	Prospective	68.1	69.3	54	54	~ 5	~ 5	—	—	KSS (K/F) 82/85	84/84	102	105	85	98
10	Ackroyd et al. [37]	2002	Prospective	70	72	408	531	6.4	5.7	St. Georg Sled	Kinematic modular	BKS (> 80 points) 77.9%	75.1%	109.3	99.9	87.5	89.6
11	Weale et al. [9]	1998	Prospective	69.6	69.8	50	52	5	5	St. Georg Sled	Kinematic modular	—	—	117.3	108	96	98

*JOA* Japanese Orthopedic Association, *BKS* Bristol Knee Scoring, *KSS (K/F)* Knee Society score (Knee score/Function score)

### Are classical ideal and nonideal indications of UKA proposed by Kozinn and Scott still valid with newer advanced prostheses?

Better surgical techniques, new implant designs, improved instrumentation, and careful patient selection have led to the higher success rates of UKA in recent years [38]. Despite being a successful treatment option for medial compartment osteoarthritis, there is an ongoing debate concerning the selection criteria for UKA, as a careful patient selection is crucial to ensure excellent long-term results. Kozinn and Scott, in their landmark article of 1987, proposed the disease- and patient-specific ideal indications for UKA surgery as the isolated medial or lateral compartment osteoarthritis/osteonecrosis of the knee, age less than 60 years, weight less than 82 kg, angular deformity less than 15° (passively correctable to the neutral), flexion contracture less than 5°, and range of motion more than 90° [39]. Patients with high activity level, exposed bone in patellofemoral compartment complaining of anterior knee pain, radiographic evidence of chondrocalcinosis, osteophytes in the opposite compartment, inflammatory arthritis, and anterior instability due to ACL insufficiency were considered nonideal candidates for partial knee replacement. Other authors have echoed this selection criteria for UKA [40]. These indications and contraindications for UKA were based on their experience only with the fixed-bearing (FB) implant and they were more intuitive than evidence based. With the advancement in prosthesis as well as surgical technique, various authors in recent studies have proposed widening the indications for UKA. They have also provided evidence of successful results with UKA performed in patients who were historically considered as "nonideal" to undergo this procedure. Pandit and colleagues, while studying 1000 patients (among them, 68% were nonideal), showed that with Oxford UKA prosthesis,10-year survival rates were similar for patients aged less than 60 years, weighing more than 82 kg, with patellofemoral arthritis, and very active patients when compared with the ideal patient group as per Kozinn and Scott criteria [41].

*UKA in patients with a high level of activity*: In the next decade, knee arthroplasty utilization is expected to grow exponentially and approximately half of these procedures will be performed in patients younger than 65 years of age [42]. The high tibial osteotomy (HTO) was previously the primary means of surgical management in younger patient population with knee osteoarthritis. However, in a study by Cross et al. comparing the conversion of UKA and HTO to TKA, the complication and reoperation rates of patients in HTO group were more than twice than those in UKA group [43].

Knee arthroplasty in young patients remains a challenge for surgeons as: (i) they are often very active with high expectations concerning the ability to return to their activities after UKA, thus remaining potentially dissatisfied even after a technically successful procedure and (ii) high and regular athletic activity leads to an increase in stress on the implant–bone interface which may potentially lead to an acceleration of poly wear and early revision. Parratte et al., in their study of FB UKA performed in 35 young patients, reported a low 80.6% survival rate [44]. Similarly, Australian [45] and Swedish [46] joint registries reported the survival rate at 7 years to be 81% in patients under 55 years of age treated with UKA. Conversely, in a recent study from Germany examining return to activity in young patients following UKA, 93% of patients returned to regular activity, and the revision rate was merely 2.5% [47]. Echoing the same, a retrospective review from Ohio done in 340 young patients aged 50 years or less showed that medial mobile-bearing UKA improved patient function and clinical parameters with a 96% survival rate free of revision surgery at 6 years and 86% at 10 years postoperatively [48]. While controversial, multiple recent studies have shown that UKA provides a viable solution for the treatment of medial compartment disease in young patient population with early patient return to function and excellent implant survival rate [41, 47, 48]. However, it is imperative that younger patients should be counseled preoperatively about potential risk for higher revision rates to set reasonable expectations from the surgery.

*UKA in obese patients* High body mass index (BMI) is a known risk factor for developing knee osteoarthritis, and trends towards a rising prevalence of high BMI and OA in younger patients have been documented. Conventionally, it is believed that obese patients undergoing UKA surgery tend to have poorer outcomes and early implant failure. Berend et al., in their series of 79 patients, reported early implant failure in 22% of cases due to persistent medial pain, tibial plateau fracture, tibial loosening, and progressive arthritis [49]. Similar outcomes were reported by Peter et al. [50] and Heck et al. [51] who reported that UKA patients with a BMI greater than 32 kg/m^2^ showed a reduced prosthesis survivorship.

As obesity has become pandemic in both developing and developed countries, a strict selection criteria based on weight is not always possible. Therefore, various studies compared the functional outcomes, survival, and complication rates of UKA in patients with normal BMI and those who were overweight and obese [52–54]. In their studies, Cavaignac [53], and Xing et al. [54] showed no adverse impact of obesity in UKA survivorship or complication rates. Tabor et al. reported higher survivorship among obese patients when compared with those who were not obese in their 20-year follow-up study of 82 patients [55]. Woo et al., in their retrospective study of 673 patients with FB UKA [56], and Molley et al., in their large prospective study of 1000 knees [57], found that high BMI is not a risk factor for loosening at a mean 10 years and there was no trend towards decreasing survival by increasing BMI. A BMI of up to 45 kg/m^2^ is proposed as cut off below which UKA can be performed with optimal outcomes [58–60]. Thus, obesity should not be considered as a contraindication for UKA surgery. However, patients with increased weight or BMI should be counseled on the preoperative risks and the conflicting evidence regarding implant survivorship and be encouraged to lose weight to help improve this modifiable risk factor.

*UKA in the presence of lateral osteophytes* Osteophytes are reported to be present in 50% of knees and are considered pathognomic of osteoarthritis; however, it is unclear whether these represent localized arthritis or are a manifestation of global arthritis. Osteophytes may develop without cartilage damage owing to joint instability or lateral joint space opening. The Kellgren–Lawrence (KL) system [61] which defines osteoarthritis as “definite osteophytes with possible joint space narrowing,” has been criticized in the past due to overemphasis on the presence of osteophytes. Progressive degeneration in the contralateral compartment is the most common reason for early failure of medial UKA resulting in early revision; therefore, the presence of osteophytes in the lateral compartment has been classically considered as an exclusion criterion for medial UKA, as this was believed to be associated with lateral side osteoarthritis. However, Waldstein et al. found in their study of 71 knees that lateral-compartment osteophytes are not associated with biomechanically weaker cartilage or with more advanced histologic signs of degeneration of lateral compartment cartilage in osteoarthritic knees with varus deformity [62]. Similarly, Faschingbauer et al., in their large series of 344 patients studied whether the presence of lateral osteophytes on plain radiographs was a predictor for quality of cartilage in the lateral compartment of patients with varus deformity and osteoarthritis (KL grade 2–3). They found no difference in the cartilage thickness or cartilage volume between knees with osteophyte grades 0–3 [63].

In their clinical outcome study, Hamilton et al. followed up their UKA patients with lateral osteophytes of different Osteoarthritis Research Society International (OARSI: atlas-based grading system ranging from grade 0—no osteophytes—to grade 3—large osteophytes) grades for 15 years. Even in knees with grade 3 lateral osteophytes, there was only one failure, with a 98% survival at 15 years [64]. The authors concluded that, in knees with full-thickness cartilage in the weight-bearing portion of the lateral compartment at the time of operation, presence or severity of lateral osteophytes do not influence long-term function or implant survival following medial UKA. So, the evidence suggests that the key to the assessment of the lateral compartment is to determine the presence of full-thickness cartilage not of lateral osteophytes. Of note, the best way to assess the full-thickness cartilage in the lateral compartment is with valgus stress radiographs done preoperatively because it is difficult to assess the cartilage thickness during medial UKA surgery. Although there are no differences in cartilage thickness and volume, it is unknown whether grade 3 lateral compartment osteophytes could cause impingement when the varus deformity is reduced by medial UKA and if the probability of full-thickness cartilage defects increases significantly with the highest grade of osteophytes (grade 3).

*UKA in patients with chondrocalcinosis (pseudogout) diagnosis*: Chondrocalcinosis is the deposition of calcium pyrophosphate dihydrate crystal in a joint. The knee joint is the commonest site for chondrocalcinosis with a prevalence ranging from 3.2% to 6.8% [65, 66]. It is generally associated with various metabolic diseases including hyper- and hypothyroidism, hemochromatosis, gout, hypophosphatasia, hypomagnesemia, and steroid therapy [67]. There seems to be a correlation between chondrocalcinosis and osteoarthritis. If seen on radiography and during the surgery, chondrocalcinosis is considered to have elements of an inflammatory arthropathy which can lead to the progression of arthritis in the other compartments and predisposes to the development of a rapidly destructive arthropathy. However, recent evidence contradicts the theory that chondrocalcinosis predisposes to progression of arthritis in the lateral compartment among UKA patients. Only 1 case among 20 UKAs with preexisting chondrocalcinosis evaluated by Woods et al. progressed to lateral compartment osteoarthritis [68]. Hernigou et al. studied 148 UKA patients with a diagnosis of chondrocalcinosis and they had no adverse outcomes [69]. Kumar et al. reported the outcome of their consecutive series of patients with chondrocalcinosis and medial compartment osteoarthritis treated with Oxford UKA prosthesis matched to controls and encountered only one failure due to disease progression in 155 cases of chondrocalcinosis with UKA [70]. Therefore, chondrocalcinosis may not be considered as a contraindication for UKA in patients with preoperative radiological evidence.

*UKA in the presence of patellofemoral joint arthritis (PFA)*: Due to the concerns that UKA results may be compromised, PFA has traditionally been viewed as a contraindication to UKA. This has prompted surgeons to preferentially perform TKA or combined UKA and patellofemoral arthroplasty (so-called bicompartmental knee arthroplasty) as an alternative. However, others have advocated for expanded criteria, providing evidence that utilization of UKA may be increased without compromising results even in the presence of PFA. Using the Altman classification [71], Berend et al. found no statistical difference in survivorship of UKA between patients with and without PFA, with 97.9% and 93.8% Kaplan–Meier survivorship at 70 months, respectively [72]. Based on their experiences with FB UKA, Thein et al. [73] and Adam et al. [74] demonstrated that preoperative patellofemoral congruence and degeneration severity without anterior knee pain do not affect postoperative functional outcomes and recommended that these patients could safely undergo FB medial UKA. Hamilton and Pandit et al. considered neither the clinical/radiological state of the PFJ nor the presence of anterior knee pain as a contraindication for UKA performed in 805 knees, except for bone loss with grooving to the lateral side [75]. In a subgroup of 100 knees, they found no relationship between functional outcomes at a mean of 10- or 15-year implant survival with the preoperative anterior knee pain, presence or degree of cartilage loss documented intraoperatively at the medial patella or trochlea, or radiographic evidence of OA in the medial side of the PFJ. Konan et al., however, found that the location of chondral lesions on the patella was an important determinant of results following UKA [76]. Centrally and laterally located chondral lesions significantly affected results and, according to the author, should be evaluated critically when considering patients with anterior knee pain and patellofemoral disease for UKA.

Patients with focal patellar PFA are indeed more likely to experience greater limitations concerning kneeling after surgery, however, we do not believe that it should be a contraindication for UKA. Firstly, though this difference has clinical implications regarding patient’s ability to kneel, these small differences do not overshadow the significant advantages that UKA provides over multicompartmental alternatives. Secondly, according to literature, patellofemoral arthroplasty and TKA both show poorer postoperative kneeling ability compared with UKA [77]. Therefore, it is unclear whether treatment with bicompartmental knee arthroplasty or TKA would actually improve kneeling ability relative to UKA in patients with medial compartment OA and medial facet patellar degeneration. Surgeons should, however, advise patients with PFA that they may experience discomfort while kneeling. Foran et al. [78] and Berger et al. [79] have demonstrated that, despite the commonality of the development of PFA amongst patients 10–15 years after UKA, the revision rate for progressive patellofemoral arthritis is only 3%. Furthermore, while the progression of arthritis in the patellofemoral compartment is common, this is rarely symptomatic. While analyzing anatomical changes following medial UKA, Thein et al. found that UKA centralizes the patellar congruence angle without impacting patellar height [73]. They proposed that restoration of patellofemoral congruence angles may release the load from the PFJ and mitigate symptoms related to patellofemoral degeneration. Therefore, patellofemoral joint osteoarthritis should not be considered as a contraindication to medial UKA, especially if the patient is asymptomatic.

In UKA, the best outcomes are achieved with proper patient selection based on its indications and contraindications. However, the above discussion derived from the plethora of evidence suggests that the thresholds proposed by Kozinn and Scott for selecting patients for UKA using weight, age, activity level, state of PFJ, and chondromalacia may not hold true. Nonetheless, if Kozinn and Scott criteria were applied, only 6–12% patients with knee osteoarthritis would be eligible for UKA; however, if expanded indications are applied, the candidacy for UKA increases to 50% [80–82]. There is an additional implication of broadening the indications of UKA as this would increase UKA usage of surgeons and as discussed earlier, could result in lower revision rates.

Goodfellow et al. recommended that indications for UKA should depend on pathoanatomy [83]. There should be substantial symptoms and anteromedial osteoarthritis/osteonecrosis, bone on bone in the medial compartment, functionally intact ACL and medial collateral ligament (MCL), and full-thickness cartilage in the lateral compartment. Some contraindications should be adhered to such as active infection, inflammatory disease, ligamentous instability, uncorrectable varus deformity, absence of the ACL, history of HTO, and severe wear of the lateral facet of the PF joint with bone loss and grooving. These refined inclusion/exclusion criteria offer the opportunity to nearly double the number of patients who fit the criteria for medial UKA, a procedure that minimizes implant costs, surgical costs, and operative times, as well as improves patient outcomes relative to bicompartmental knee arthroplasty and TKA.

### Preoperative MRI: Do they have a role in the decision-making while considering UKA in a patient?

Several radiographic studies are recommended for planning UKA including weight-bearing long-leg anteroposterior (AP) view, Rosenberg, merchant, lateral projections, and stress views. Some surgeons also prefer MRI to evaluate ligamentous structures and the lateral and patellofemoral compartment cartilage status before surgery. However, the role of preoperative MRI is debatable as it sometimes overestimates the degree of knee pathology. Disler et al. reported that nearly two-thirds of all routine knee MRIs demonstrated articular cartilage damage of uncertain clinical significance [84]. Sharpe et al. reported that, while 33% of patients with anteromedial osteoarthritis had a degenerate ACL according to MRI, only 13% patients had deficient ACL on surgical inspection. They concluded that MRI was too sensitive to be of “any practical value” in evaluating the ACL because its structure and function in osteoarthritis is highly variable [85]. In addition to the questionable utility, a routine MRI scan for planning UKA may be an avoidable expenditure [86]. Moreover, abnormal preoperative MRI findings may not influence the outcome of UKA when modern radiographic and clinical criteria are met with the appropriate intraoperative assessment. Hurst et al. reviewed 33 UKA patients who had preoperative MRI with interpretations of osteoarthritic changes in the lateral compartment, patellofemoral compartment, and/or deficiency of the ACL and reported excellent functional outcomes with only 3% failure rate [87].

Instead of a routine preoperative MRI scan, surgeons could effectively utilize physical examination for ligaments and radiographic study. Accordingly, patients with substantial ACL laxity, lateral patellar facet grooving, or collapse of the lateral compartment on valgus stress view (> 5 mm narrowing of lateral joint) should not be offered UKA. Besides, UKA should be abandoned on behalf of TKA if intraoperative inspection reveals ACL incompetence or a full-thickness weight-bearing articular cartilage lesion on the lateral femoral condyle. However, surgeon reliance on the test of normal laxity of the ligament is open to error, for no quantitative measure can be reliably applied to the stretching of a ligament at surgery. Loss of height of the joint due to hyaline articular cartilage destruction leads to increased laxity of the ACL, which may in fact function normally once the articular height is restored by arthroplasty. Conversely, osteophyte impingement may stretch a lax ligament giving the false impression of normal tension on testing the ACL by the Lachman test. Thus, a preoperative MRI is not routinely necessary before UKA; however, when the clinical presentation is not clear, an MRI can be very useful in assessing other conditions such as avascular necrosis and neoplasm which otherwise might have gone undetected.

### Errors in component placement: Should incision be minimal or optimal?

A surgical technique from its incision to the closure of the wound should be precise, safe, accurate, and reproducible. Consequently, it becomes very pertinent for any surgeon to develop evidence-based surgical protocols for favorable outcomes. Incision and exposure, the very first step of surgery, plays a vital role not only during surgery but also in rehabilitation post surgery. Conventionally, a medial parapatellar arthrotomy incision is used by most knee arthroplasty surgeons for both TKA and UKA, although the prolongation of incision proximally may be variable. Extension of the incision can be through the quadriceps tendon or the vastus medialis muscle, or a subvastus approach can be contemplated. Each approach has its advantages and disadvantages but patellar eversion is the common step which has been considered harmful. The advantages of a minimally invasive surgery (MIS) that have been reported as a reduced need for postoperative pain medication, more rapid return of knee flexion and functional activities as compared to the conventional medial parapatellar approach. Therefore, a minimally invasive incision was proposed for UKA surgery which could particularly avoid the undesirable patellar eversion. This minimally invasive approach has variations with highlights and challenges: (a) Quadriceps sparing technique with only a medial parapatellar arthrotomy without any proximal extension into the quadriceps; (b) Midvastus technique in which vastus medialis is incised for 2 cm along with medial parapatellar arthrotomy; and (c) Subvastus approach in which the medial parapatellar incision is prolonged by elevating the distal part of the vastus medialis muscle without performing any musculotendinous incision [88].

Haas et al. reported more rapid functional recovery with improved range of motion in TKA done with a minimidvastus approach without any malposition of implants with both standard open and mini-open surgery [89]. Price and colleagues reported the average rate of recovery after short-incision UKA to be twice as fast as that of standard-incision UKA without any significant difference in implant positioning [90]. Significant reduction in hospital bed occupancy and accelerated discharge with rapid rehabilitation resulted in 27% cost saving when mini-open surgery was compared with standard-incision UKA in a study by Reilly et al. [91]. However, some studies have raised a concern about the loss of accuracy while using the minimally invasive approach for UKA. For instance, Muller et al. comparing the two techniques in UKA surgery, found superior functional outcomes but suboptimally positioned implants with MIS than conventional incision [92]. Similarly, Dalary and Dennis showed that a significant number of patients had tibial component varus malalignment in mini-open UKA [93]. Berend and colleagues reported a 20% failure rate in short-incision UKA after follow-up of 3 years [49]. A study by Hamilton et al. observed a high revision rate and more frequent aseptic loosening for minimally invasive UKA compared with conventional UKA [94].

However, from the above discussion, it is still not clear whether to perform a minimally invasive approach or not, as the evidence is contradictory. Pragmatically, the choice of incision should depend on the preference of the surgeon and his/her expertise, as the “size” is an individual perspective. Whichever incision is chosen, this should not violate the principles of a surgical approach. Moreover, due to an increase in the life expentancy accros the globe, the probability of revision after UKA is high. Therefore, the incision should be such that future revision is comfortable. Exposure should be such that the proper landmarks for bony cuts and soft-tissue balancing are easily localized. There should be no struggle during surgery in any step as soft-tissue manhandling may inadvertently lead to poor results, which would defeat the very purpose of MIS. Complete evaluation of ACL sufficiency and the other compartment arthritis should be possible with the incision, and the surgeon should be able to work comfortably for the accomplishment of proper and complete tibial and femoral cuts with trouble-free cementation.

### Choice of bearing design: Mobile or fixed bearing?

Since the introduction of UKA more than 30 years ago, the prosthesis design has significantly modified resulting in excellent functional outcomes and increased survivability. Similar to TKA, UKA surgery also has options of FB as well as MB polyethelyne designs. However, the choice of bearing design is thought to influence the functional outcomes and longevity of UKA quiet differently than in TKA. Historically, the first available UKAs were cemented FB all-polyethylene UKAs. However, due to the increased incidence of polyethylene wear, metal-backed tibia with FB polyethylene was developed. Subsequently, in 1986, Goodfellow and Connor introduced a MB UKA design to further improve upon the wear characteristic [5]. Early retrieval analysis supported this notion by showing low wear rates with a fully conforming MB design [95]. Each has its advantages and disadvantages, although choosing the appropriate bearing type for UKA surgery remains somewhat controversial [96].

**Fig. 2:**
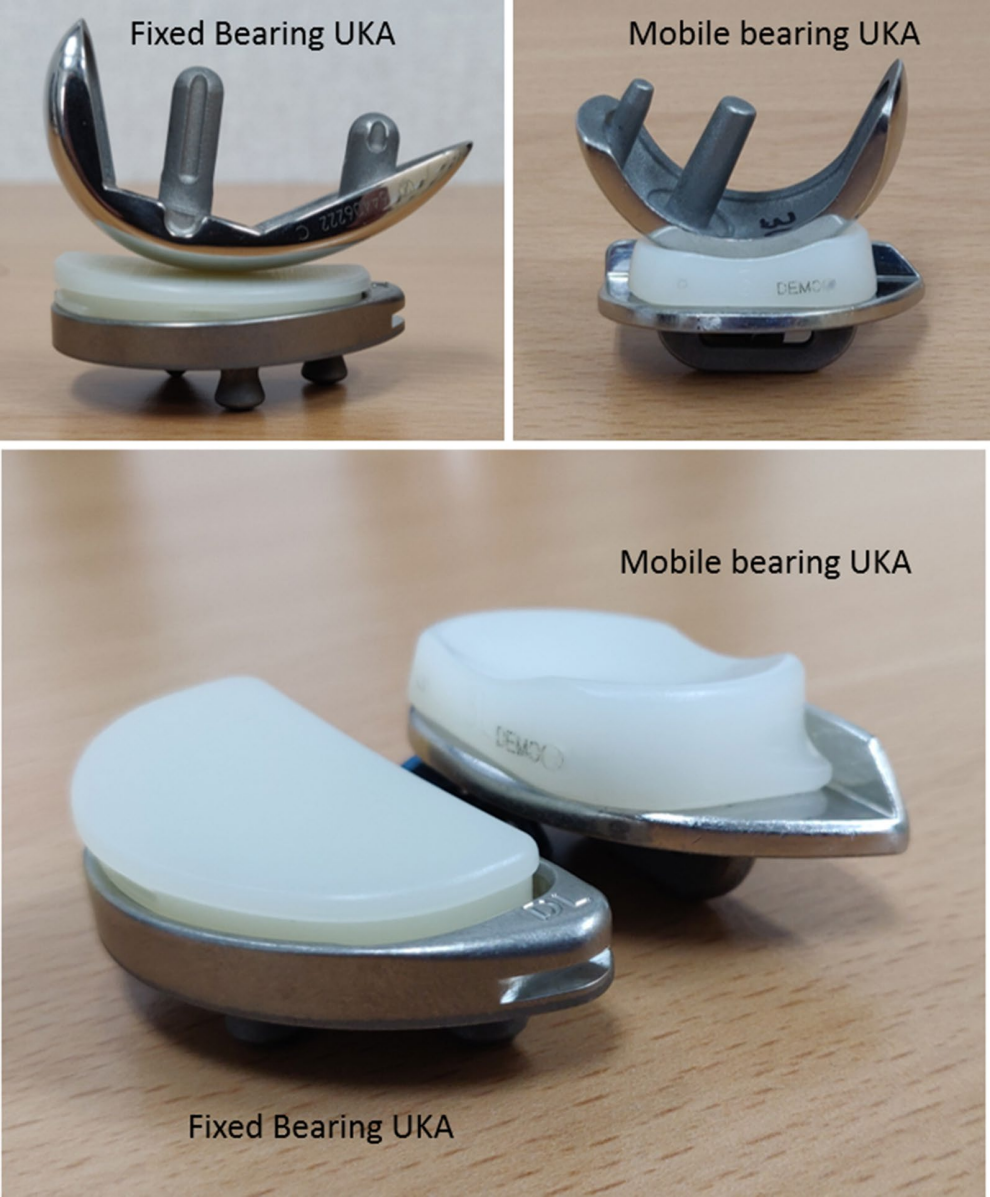
Fixed bearing UKA: noncongruous articular surface, small contact area, large point contact force, and ‘insert’ fixed to base plate. Mobile bearing UKA: congruous articular surface, large contact area, small point contact force, and mobile ‘insert’

These two designs are different in their fundamental concept [97] (Fig. 2) and in the surgical techniques required for their implantation. The MB is designed to allow more natural joint kinematics [98, 99], while also allowing a higher degree of conformity between the articular surfaces. Its distinctive design is proposed to reduce the surface and subsurface contact stresses granting less likelihood of polyethylene wear compared with FB designs [100, 101]. An intact healthy ACL is a prerequisite for MB UKA, as there is increased stress on the ligaments. A high incidence of rupture of degenerated ACL and MCL has been reported with MB poly which results in instability of the knee [102]. Besides, MB design has a peculiar concern of bearing dislocation which can ultimately lead to early failure. The rate of bearing dislocation ranges from 0.6% to 6.5% in different studies [101, 103]. Overrelease of medial collateral ligament, undersized bearing, component malposition, and flexion–extension imbalance are some of the causes of bearing dislocation [104]. Consequently, surgeons tend to keep the medial joint space of the knee tight so that bearing does not slip out. Furthermore, the surgeons tend to perfom valgus overcorrection to offload the medial compartment and further decreasing the probability of bearing dislocation and aseptic loosening [105]. Failure to do so meticulously may lead to undesirable complications of accelerated progressive lateral compartment osteoarthritis. This occurs especially when surgeons fearing bearing dislocation overcorrects the deformity into valgus alignment, leading to overt contact stresses on the lateral side [106, 107]. Conversely, if the varus deformity is undercorrected, the chances of bearing dislocation and aseptic loosening increases [108]. Therefore, in MB design, the precise alignment and ligamentous balancing are essential to prevent bearing dislocation or impingement, which predisposes this design to be more prone to surgeon-related errors. The revision rate of MB prosthesis is related to UKA usage as well as the annual operation volume of the surgeon because experienced surgeons are more meticulous in maintaining the ideal surgical parameter required for pristine UKA surgery [109, 110].

On the other side of the spectrum is FB design. They have less conforming articular surfaces with only micromotions between the tibial baseplate and polyethylene insert. Although this type of geometry increases the point loading, there is less risk of bearing dislocation than the MB prosthesis. A randomized  controlled study by Li et al. upon the in vivo kinematics of UKA did not demonstrate the proposed kinematic advantage of MB over FB, probably due to the fact that both cruciates are retained in UKA [111]. Due to higher contact stresses caused by relatively flat articular insert, the polyethylene wear rate is higher in FB than MB UKA designs [95]. However, Burton et al., in an in vitro study comparing wear rates of MB and FB designs showed reduced cumulative wear with FB UKA [112]. They found that, in both the designs, the lateral side had an increased amount of wear, suggesting that increased motion on the lateral side plays a larger role in wear generation than increased weight-bearing as seen medially.

As suggested by Zuiderbaan et al., slight varus undercorrection is desirable for optimal results in UKA surgery. As there is less likelihood of bearing dislocation in FB, undercorrection can be attempted with these designs [113]. At the same time, avoiding overcorrection offloads the lateral compartment and may decelerate its progressive arthritis. Unlike MB designs, FB prosthesis allows loose medial joint space in UKA, which decreases the risk of aseptic loosening. At the end of FB UKA surgery, a 2-mm laxity during the valgus stress at 20° flexion is the goal. The higher technical requirements and the longer learning curve needed for the surgeon in MB design is not required in FB designs, thereby making it more tolerant to surgeon-related errors and more friendly for less experienced surgeons. Regarding the incidence of radiolucent lines, studies have demonstrated an increased frequency of nonprogressive radiolucencies in MB UKA than FB design, probably because of micromotion between implant, cement surface, or both and the bone or due to overtension of the revised compartment [111]. This leads to higher chances of misinterpretation of physiological radiolucent lines resulting in erroneous revision in otherwise well-fixed UKA. While MB designs fail early due to bearing dislocation and aseptic loosening, FB design tends to fail late due to polyethylene wear and osteoarthritis progression in the non-replaced compartment [108]. Neufield et al. reported 83% 10-year survival of the MB compared with 90% survival for FB UKA design. Unlike FB UKA, the revision of MB design required stems or tibial augments making the reoperation more difficult [114]. Similarly, Bloom et al. reported that 46.7% of the MB UKA revisions required tibial augments compared with only 11.1% in the FB group [115]. MB designs can be more technically challenging, with a more pronounced learning curve, which can lead to the variability in results seen in literature, especially in studies including heterogeneous high- and low-volume centers. Bonutti et al. recommended that, low-volume surgeons are more likely to achieve predictable and high rates of survival using FB design for UKA [97].

Despite the difference in design and their mechanics, clinical outcomes and revision rates of FB and MB UKA done in primary UKA are reported as equal. Swedish [116] and Finnish [117] arthroplasty registries comparing both bearing designs of UKA suggested no conclusive advantage of one bearing design over the other in terms of prosthesis survivorship. Winnock de Grave et al. assessed 460 FB UKA and showed 94.2% 10-year survivorship and excellent or good outcomes in 94.6% of patients [118]. Similarly, 825 MB UKA were assessed by Alnachoukati, who reported 90% 10-year survival with a mean Knee Society score of 90 postoperatively [119]. A metaanalysis by Peersman et al. also showed no differences between the two designs in terms of functional results [96]. Furthermore, a minimum 15-year follow-up long-term study by Parratte et al. observed no difference in mean Knee Society scores and survivorship between the two bearing designs [120]. A systematic review of 15 studies comparing FB and MB UKA found that there was no difference in revision rates, complications, or knee function [121]. The treatment option should be carefully considered for each patient and the surgeons should use their personal experience while deciding between these two bearing options.

### Should we use cementless implants instead of cemented ones?

Similar to TKA, UKA can be implanted with the use of cement or using a porous-coated prosthesis surface without cement. Currently, most knee arthroplasty surgeons consider cemented UKA as gold standard despite availability of cementless implants and instrumentation, including porous-coated stem, hydroxyapatite stem fixation, and modified designs for the past 20 years. Conventional training of surgeons with cemented implants, and the high failure rates reported by a study by Lindstrand in the initial use of cementless UKA are possible reasons for underuse of cementless UKA [122].

Cementless implants have several proposed advantages over cemented implants [123, 124]. The fixation strength in cemented prosthesis depends upon the bone ingrowth, which can lead to reliable fixation, especially in young patients. Uncemented implants eliminate the errors associated with cementation, reduced impingement [125], and no cement particulate debris which can lead to accelerated wear [126], shorter operative time, and low incidence of misinterpretation of radiolucent lines (RLLs) [127]. RLLs are prognostic factors for making a diagnosis of aseptic loosening and can be physiological or pathological. Physiological RLLs are well defined, 1–2-mm thick, and accompanied by a radiodense line [128], in contrast to pathological RLLs that are > 2-mm thick, poorly defined, and have no radiodense line [129]. Pandit et al. found that radiolucency occurs less frequently in uncemented UKA (6.3% versus 75%) in cemented UKA [127]. It has been argued that cemented UKAs show a higher incidence of radiolucencies because of the  possible incomplete cementation, thermal osteonecrosis, and formation of fibrous tissue [130, 131]. Liddle et al. showed that the physiological radiolucencies are often misinterpreted on radiographs [126]. The authors defined these radiolucencies as narrow, nonprogressive, and representative of an incomplete fibrocartilage layer that does not negatively impact implant survival. In the Oxford medial UKA, the vertical wall of the tibial component is not coated with porous titanium and therefore often has adjacent radiolucencies when evaluated on radiographs postoperatively, which can be safely ignored. Thus, presence of RLLs may lead to a higher incidence of misinterpretation in cemented UKA compared with cementless prosthesis resulting in erroneous implant revisions in otherwise well-fixed and good-functioning arthroplasty.

UKA is proposed to be more befitting for cementless fixation than TKA because of the mechanical advantage of UKA at the bone–implant interface. There are mainly compressive loads both when the knee is centrally and eccentrically loaded with UKA which is an ideal condition for achieving osseous ingrowth with cementless fixation [132]. Also, shearing stress and tilting are minimal due to the absence of tibiofemoral constraints, especially in MB UKA. Liddle et al. further suggested that soft-tissue releases performed during routine TKA require increased tibiofemoral constraint in the form of a cam-and-post mechanism or dished polyethylene, which increases the shear forces imparted to the implant–bone interface and predisposes the prosthesis to aseptic loosening [126].

Daniilidis et al. while retrospectively studying 106 knees (42 cemented and 64 uncemented UKA), showed significantly better quality of life in cementless-implant patients but, at the same time, reported more and larger periprosthetic loosening areas in the cementless tibial side on radiological analysis [133]. Forsythe et al. also echoed similar concerns regarding tibial side radiolucency in cementless implants [134]. Despite early conflicting results, in the last 10 years, several specialist centers have showed encouraging clinical outcomes and survival of modern implants. Kendrick et al. in a RCT using radio isometric analysis showed no complete radiolucencies with uncemented implants, whereas 24% of cemented UKAs had complete RLLs. They concluded that the function of cementless-implant component is at least as good as if not better than that of cemented devices [124]. Pandit et al. showed no difference in clinical outcomes between the two and showed narrow RLLs at the bone–implant interfaces in 75% of cemented tibial components, whereas in the cementless implants, there was no complete radiolucency and only 7% partial lucency [127]. This implied satisfactory bone ingrowth into the cementless implants. Campi and Pandit, in their systematic review of 10 papers (1199 knees) on cementless fixation in UKA found that the 5-year survival ranged from 90% to 99% and the 10-year survival from 92% to 97% and that the progression of osteoarthritis in the remaining compartment was the most common cause of failure [135]. Clinical outcomes, failures, reoperation rates, and survival were comparable to those reported in similar studies on cemented UKAs. This review also suggested that the robotic UKA may facilitate the implementation of cementless implants for future UKA prosthesis designs. Recent design developments, including utilizing porous titanium surfaces that allow for osseous ingrowth and coating the prosthesis with biologically active materials such as hydroxyapatite have demonstrated improved clinical and radiographic outcomes but with extra cost. Nonetheless, we need adequately powered RCTs and longer follow-up periods comparing cemented and cementless UKA components for reaching on a conclusive decision about superiority of clinical outcomes including survivorship.

### Ideal limb alignment and optimal position of UKA prosthesis: What is the consensus?

**Fig. 3:**
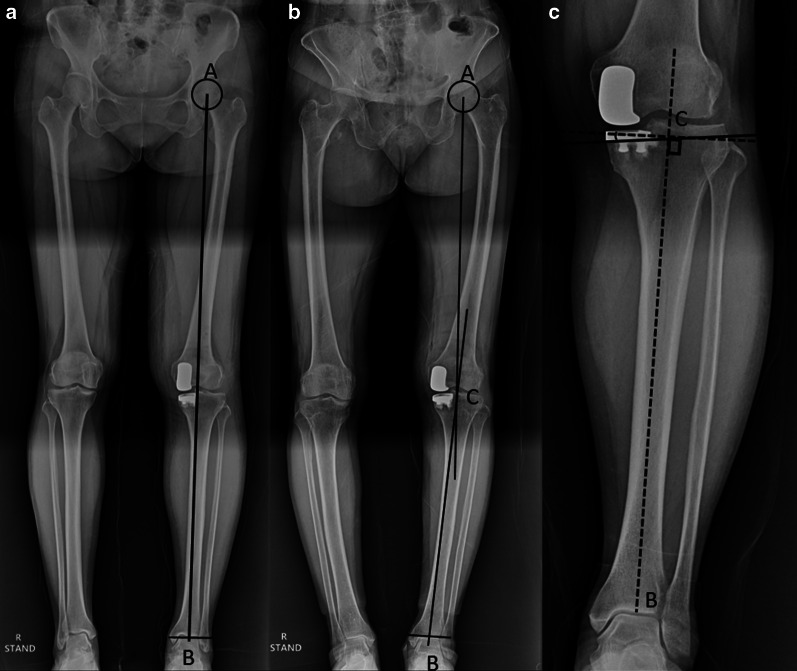
**a** Mechanical axis (Maquet’s line) is a line from center of femoral head (A) to center of ankle (B); **b** Hip-knee-ankle axis is the angle between mechanical axis of femur (A to center of knee, C) and mechanical axis of tibia (C to B); **c** Tibial component coronal alignment is the angle between the line perpendicular to the mechanical axis of tibia and coronal axis of tibial component

**Fig. 4:**
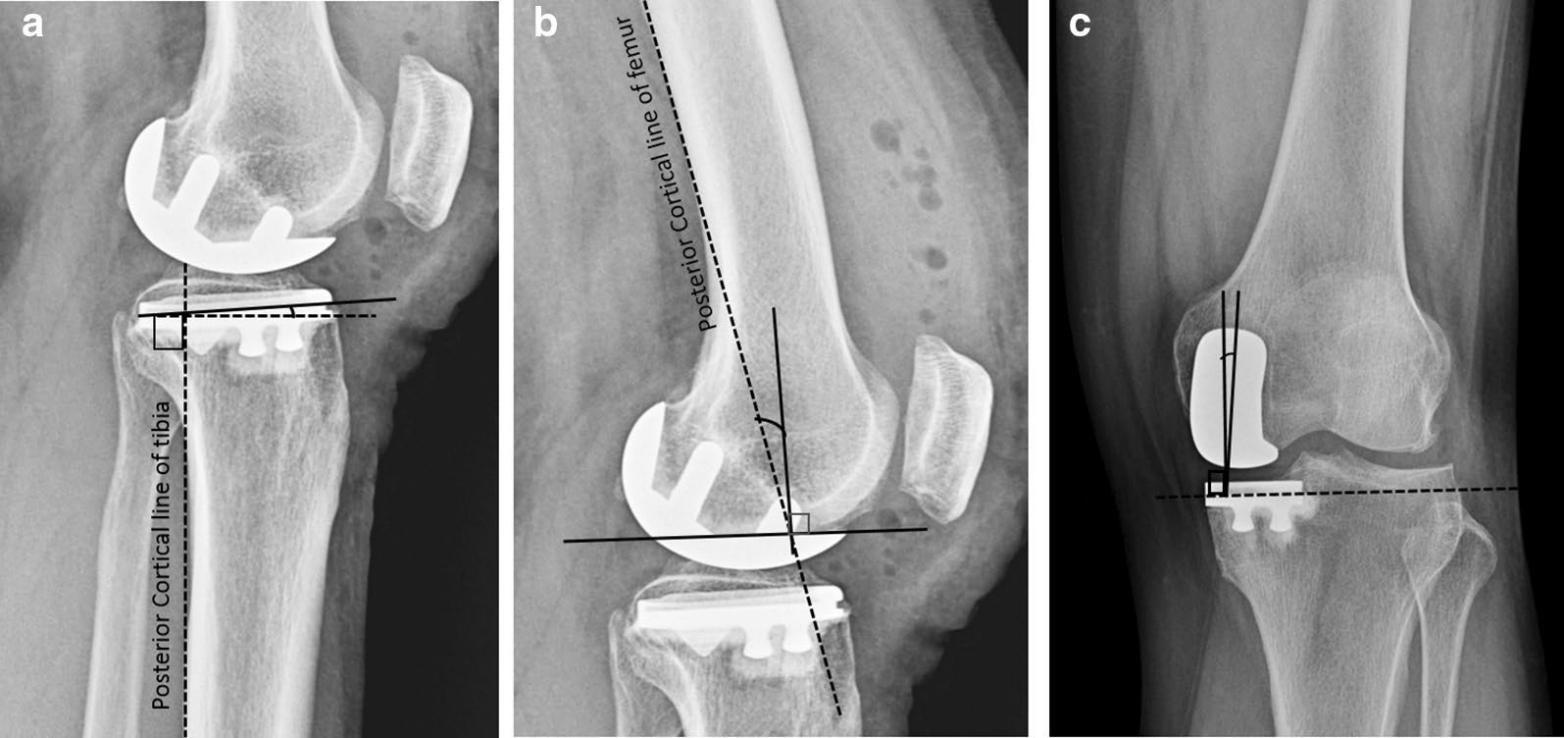
**a** Tibial component slope is determined by the angle between the line perpendicular to posterior tibial cortex and the tibial component sagittal axis; **b** Femoral component sagittal alignment is determined by the angle between the line perpendicular to the component part placed on distal femur cut and the posterior cortical line of femur; **c** Interprosthetic divergence is the angle between the line perpendicular to the tibial component coronal axis and the long axis of femoral component

UKA must be aimed to restore pre-osteoarthritic femorotibial geometry accurately. Several parameters have been identified as responsible for maintaining normal knee kinematics and thus avoiding common complications such as knee pain, polyethylene wear, and lateral compartment accelerated osteoarthritis in UKA. Limb alignment, component position and sizing, ligamentous and soft-tissue balancing, interprosthetic divergence, and maintenance of the inherent joint line are some of the surgical variables which have been shown to influence the clinical outcomes of UKA as well as its longevity [136] (Figs. 3, 4).

*Limb alignment*: The hip-knee-ankle axis (Maquet’s line) determines the limb alignment and is a major contributor to a successful UKA [137]. While in TKA a neutral mechanical axis of 0° is recommended, in UKA, the surgeon should correct only the part of the deformity that resulted due to wear, thus restoring the original (predisease) mechanical axis for appropriate ligament tension. Postoperative alignment in UKA is dependent on the thickness of the tibial implant, level of resection of the tibia, ligamentous balance, and preoperative deformity. Varus/valgus inclination of UKA components does not affect the lower-limb alignment but only the obliquity of the joint line. Hernigou and Deschamps while studying medial UKA, found that severe undercorrection of varus deformity could lead to accelerated polyethylene wear with early implant loosening, and at the same time, an overcorrection to valgus was found to result in rapid degeneration in the lateral compartment [138]. Zuiderbaan et al. suggested that a postoperative varus angle of 1–4° should be pursued while performing medial UKA for obtaining superior functional results [139]. Similarly, Chatellard and colleagues reported high rates of mechanical failure with residual varus of 5° or more [25]. Vasso and colleagues studied 125 medial FB UKA with no more than 7° varus limb alignment and observed that minor varus alignment does not compromise the mid- to long-term outcomes of medial UKA and achieves better results compared with neutral alignment [140]. In general, neutral limb alignment produces good outcomes, but slight undercorrection of the initial deformity may result in the more favorable outcomes. According to few studies, a hip-knee-ankle (HKA) angle of 1–4° varus optimizes subjective results of WOMAC domains of pain, function, and total scores compared with an HKA angle < 1° or > 4° [139, 140].

*Knee joint line*: Restoration of the joint line in both medial and lateral compartments appears to play a role in successful outcomes following UKA. The height of the prosthetic joint space affects load transfer between the two femorotibial compartments. Finite element analysis in a previous study demonstrated increased contact stress on both the polyethylene insert and articular cartilage with a more than 6-mm change in joint line [141]. Using a validated software model for measuring joint congruence, Khamaisy et al. demonstrated that well-maintained joint line in medial UKA improves the congruence and joint space width of the lateral compartment [142]. Elevating the medial joint line more than 5 mm has also been shown to result in loss of extension (flexion contracture) after UKA [143]. Mazas found that 54% of the load is generated through medial UKA when joint space lowering is associated with 5° of undercorrection. In contrast, joint space elevation associated with 5° of overcorrection transferred 88% of the load to the lateral femorotibial compartment [144]. He recommended that the prosthetic joint space height should be within 3 mm in either direction of lateral compartment joint space height to restore balance between the two femorotibial compartments. Besides, if the transverse tibial cut is placed too distally, the tibial implant rests on cancellous bone, which offers less resistance to compressive forces, as demonstrated in an experimental study by Lesaka et al. [145]. Restoring joint space height is therefore crucial in terms of both joint mechanics and joint kinematics, as this is also responsible for limb alignment.

*Tibial component position*: The positioning of the prosthesis in different planes has been shown to affect the functional outcomes and the survivorship of UKA, especially the tibial component placement. In a retrospective multicentric study of 559 medial UKA, Chatellard et al. found that the tibial component obliquity is an issue that affects joint kinematics restoration and bone resistance to loading. They indicated that the physiological obliquity of the femorotibial joint space which is about 3° of varus, should be restored to within 3° in either direction [25]. Using radiostereometric analysis, Barbadoro et al. found that varus angulation of the tibial component > 5° resulted in increased implant micromotion that could lead to loosening [146]. Collier et al. studied 245 FB UKAs and found that leaving the medial tibial plateau with varus angulation resulted in higher failure rates [147]. A normal tibial slope should be restored, as slope influences both bone quality and knee kinematics. A greater change in slope value affects the flexion range of the prosthetic knee. An excessive slope results in active anterior tibial translation, placing excessive load on the ACL. Subsequent distension of this ligament may result in knee instability, whereas the absence of ligament distension with load transfer to the prosthetic plateau may result in tibial-component loosening. Hernigou and Deschamps retrospectively reviewed 99 UKAs with an average follow-up of 16 years and found posterior tibial slope > 7° to be associated with a higher risk of loosening [148]. Chatellard et al. identified two criteria: absolute slope should not exceed 5° and the change in slope should not be greater than 2° relative to the physiological value for increased longevity of UKA [25]. Sizing of the tibial component appears to matter as well. Tibial components with > 3 mm overhang were found to have significantly worse Oxford knee scores at 5 years after surgery, but there was no difference between implants with < 3 mm overhang compared with undersized components [149]. However, the authors still cautioned against significant undersizing because of the risk of subsidence and loosening.

*Interprosthetic divergence*: In addition to the above-described criteria for tibial component positioning, the relationship between tibial and femoral components also plays a role in UKA survivorship. Divergence is influenced by both implant position and implant geometry as shown in studies of Scandinavian registries. Both components should form a 90° angle with each other. Chatellard et al. indicated a margin of tolerance of 6° in either direction, and they believed that intraprosthetic implant alignment depended chiefly on prosthesis design [25]. Therefore, alignment issues should be discussed in the recommendations developed by manufacturers.

### Role of ACL deficiency in UKA; ACL reconstruction simultaneously with UKA: Is it too much?

The role of ACL functional integrity has been debated among UKA surgeons [150]. The traditional opinion is that a functionally intact ACL is a fundamental prerequisite to perform UKA [151]. Both traumatic ACL rupture and degenerative ACL deficiency can predispose to osteoarthritis in the primary knee. Patients with primary ACL rupture generally present with pain and instability and develop secondary posteromedial OA [152]. This group of young and active patients should not ideally be considered candidates for isolated MB UKA because of the increased risk of failure from bearing dislocation, polyethylene wear and tibial loosening due to knee instability [153]. This is possibly related to the eccentric loading of the tibia in ACL-deficient knees. When the ACL was deficient, higher failure rates were reported with MB implants [102] and with the Lotus implant [153], a relatively flat FB component. Goodfellow et al. in their 103 MB UKA study found a 21% failure rate within the first 2 years of implantation in ACL-deficient knees [102]. In contrast, the absence of an ACL did not lead to failure with the St. Georg and Marmor implants [151].

Elderly patients who develop secondary degenerative ACL functional deficiency due to primary anteromedial OA are nowadays accepted as possible candidates for UKA. Biomechanical data suggest that leveling of the tibial slope may compensate for anterior translation in the ACL-deficient knee without restoring the pivot shift to normal [154]. Also, the functional requests or the presence of posterior osteophytes and capsule stiffness prevents elderly patients from instability symptoms in most cases. Recent papers have begun to confirm good short- to mid-term outcomes without increased risk of prosthesis failure in this population. Boissonneault et al. in their small series of 46 patients with short-term follow-up and mean age of 65 years reported the 5-year survival rate for medial UKAs in ACL deficient knees to be 94% which was comparable to UKAs in ACL-intact knees [155]. ACL insufficiency being considered an absolute contraindication for UKA is debatable especially for newer FB implants as long as the degenerative pattern on the tibiofemoral joint is anterior. Notably, it is critical to minimize the tibial slope in ACL-deficient knees.

A special subgroup of patients is young individuals with rotatory ACL instability in combination with anteromedial knee OA. Technical advances and the widening of surgical indications have culminated in the advent of combined UKA and ACL reconstruction surgery which has shown promising results [156]. A review by Weston-Simons reports 93% implant survival at 5 years for 52 patients with a mean age of 51 years who underwent staged or simultaneous ACL reconstruction and MB UKA [157]. Combined UKA and ACL reconstruction is a longer and more technically demanding procedure but avoids the need for reoperation associated with one more anesthesia, longer recovery time, and higher social costs. A staged procedure starting with ACL reconstruction may be indicated if instability is the main symptom, proceeding with UKA only if pain arises later.

Theoretically, an absent ACL would increase the sliding motion that caused accelerated polyethylene wear of UKA in laboratory studies. However, through careful patient screening, altered intraoperative technique, and concurrent ligament reconstruction, patients with ACL-deficient knees may benefit from UKA in knees with isolated single-compartment disease. In the authors’ opinion, FB UKA without ACL reconstruction can be a wiser choice in ACL-deficient knees in the low-demand young population as well as in elderly patients. Besides, performing combined ACL reconstruction with UKA is cumbersome, requires expertise, and may result in suboptimal outcomes because of still unknown complications of performing ACL reconstruction simultaneously with UKA.

### Is UKA a cost-effective surgery?

Joint replacement procedures including knee arthroplasty are among the most expensive procedures that are regularly performed among health-insurance beneficiaries. However, economic evaluation of any surgical procedure, if expressed in monetary terms only, would be an unfair practice. This should involve both the cost and quality-adjusted life years in the final evaluation. Therefore, effectiveness of a treatment modality should be measured in terms of (a) cost-effectiveness analysis (a common unit of clinical effect), (b) cost-utility analysis (a generic measure of health gain), and (c) cost–benefit analysis (a monetary measure) [158].

The cost of surgery and subsequent rehabilitation and reoperation is borne by either insurance company or by the patient himself depending upon the country and their health schemes. In any case, the burden of the high cost of any procedure is detrimental to the patient as well as for their country’s economy. Hence, it becomes pertinent that surgeons follow a procedure that is cost-effective and at the same time, associated with satisfactory functional outcomes. UKA has been associated with a high revision rate, which can offset the initial cost–benefit gained by a shorter hospital stay and earlier rehabilitation. However, trade-offs between upfront benefits and later risk of revision of UKA compared with those of TKA are poorly understood. Konopka and his colleagues in 2008 followed up 50,493 knee replacement patients identified from the Finnish arthroplasty register and recommended discontinuation of UKA in unicompartmental OA based on low-cost effectiveness owing to high revision rates [159]. However, the analysis was not comprehensive and the study design had several methodological limitations. In contrast, recent literature without any uncertainty reports that UKA is a cost-effective procedure and should be contemplated when indicated [160, 161]. Many authors have attempted cost-effective analysis (Table 2); some are based on their literature review and their cohort analysis, and others are based on the registry data of different countries [13, 17, 159, 161–169].

**Table 2:** Summary of studies comparing cost effectiveness of UKA and TKA

S. no.	Author	Year	Type of study	Country	Mean age (years)		Number of cases		Mean hospital stay (days)		Cost effectiveness/comments
Based on registry data					UKA	TKA	UKA	TKA	UKA	TKA	
1	Ghomrawi et al. [162]	2015	Markov decision model	Swedish registry	45–85	45–85	—	—	—	—	35,000 (85 years) to 46,600 (45 years) USD for UKA versus 42,000 to 47,600 USD for TKA
2	Peersman et al. [163]	2014	Markov decision model	Belgian registry	—	—	—	—	3	5	UKA 2807 Euro less costly than TKA
3	Willis-Owen et al. [17]	2009	Retrospective	UK registry	—	—	—	—	5.9	8.3	UKA 1761 Euro less costly compared with TKA
4	Koskinen et al. [13]	2008	Retrospective	Finnish registry	65	70	1886	48,607	8.5	10.4	Costs saved by lower implant prices and shorter hospital stay for UKA would not cover costs of extra revisions.
5	Slover et al. [164]	2006	Markov decision model	Norwegian registry	—	—	—	—	—	—	UKA 200 USD less costly than TKA
6	Robertson et al. [165]	1999	Retrospective	Swedish registry	—	—	10,624	15,437	10.7	12.3	UKA 1645 USD less costly than TKA
Based on literature review											
7	Konopka et al. [159]	2015	Markov decision model	USA	—	—	—	—	—	—	24,636 USD total medical direct cost for UKA compared with 24,761 USD for TKA
8	Smith et al. [166]	2014	Markov decision model	UK	70	70	—	—	—	—	5235 Euro for UKA versus 6805 Euro for TKA
9	SooHoo et al. [161]	2006	Markov decision model	USA	—	—	—	—	—	—	2562 USD less lifetime treatment cost with UKA compared with TKA
Based on within-study analysis data											
10	Shankar et al. [167]	2015	Prospective	USA	63.9	63.9	64	64	2.3	3.8	11,397 USD for UKA versus 16,243 USD for TKA
11	Xie et al. [168]	2008	Prospective	Singapore	63	67	102	431	5.3	7.7	6824 USD for UKA versus 8513 USD for TKA
12	Yang et al. [169]	2003	Prospective	Singapore	65.1	65.5	50	50	3.6	6.9	8700 USD for UKA versus 12,000 USD for TKA (total hospital bill)

The cost-effectiveness of UKA can be affected either by patient factors or the surgeon related factors. Among patient factors, age is an important variable in UKA cost–benefit analysis. Burn and Liddle in their population-based study using data from NJR from England and Wales found that the largest expected savings were for males over 75 years, while the biggest improvement in the quality of life was for females over 75 years [158]. However, it is less certain and highly variable for younger patients who have a higher risk of lifetime reoperations and revision. The variation in findings for younger patients across studies appears to be driven by differences in the estimates for both the risk of revision and the expected effect of revision on quality of life. However, modest improvements in implant survivorship could make it a cost-effective alternative in younger patients. Among surgeon related factors, surgical caseload as well as UKA usage are important considerations in the analysis of cost-effectiveness [170]. When UKA was performed by surgeons with usage above 10%, UKA was found to be unequivocally cost-saving and health-improving compared with TKA. When performed by surgeons with usage less than 10%, however, UKA was no longer expected to lead to better health outcomes than TKA and TKA became the more likely cost-effective procedure [171]. UKA still compares favorably in economic evaluations of estimated cost and health outcomes even when considering slightly higher rates of revision.

### Robotics, computer navigation, and patient-specific instrumentation and implants: conventional versus technology-assisted UKA

UKA, despite being associated with excellent outcomes and 96% chances of return to preoperative activity level [172], is contemplated only in 5–10% knee arthroplasty surgeries. In contrast, TKA, which has a dissatisfaction rate of ~ 14–19% [173] is still being ubiquitously used. UKA is a challenging procedure and outcomes after UKA patients are less tolerant to surgical errors. Execution of surgical plan intraoperatively thus becomes very important; otherwise, this may be counterproductive, as more surgeon-controlled variables have been linked to the survival of UKA. Chatellard et al. observed that a high level of accuracy is required for the optimal position of the implant and that even minute changes in the position can lead to the revision of UKA [25]. Therefore, to improve functional outcomes and address the inconsistent durability of UKA, surgeon-controlled errors must be minimum along with least outliers. There might also be a concern about the loss of accuracy with minimally invasive techniques. Consequently, assistive technologies have been developed with the potential and claims to improve the accuracy of implant positioning and limb alignment even with a minimally invasive technique by minimizing surgeon-controlled errors using smart tools.

**Fig. 5:**
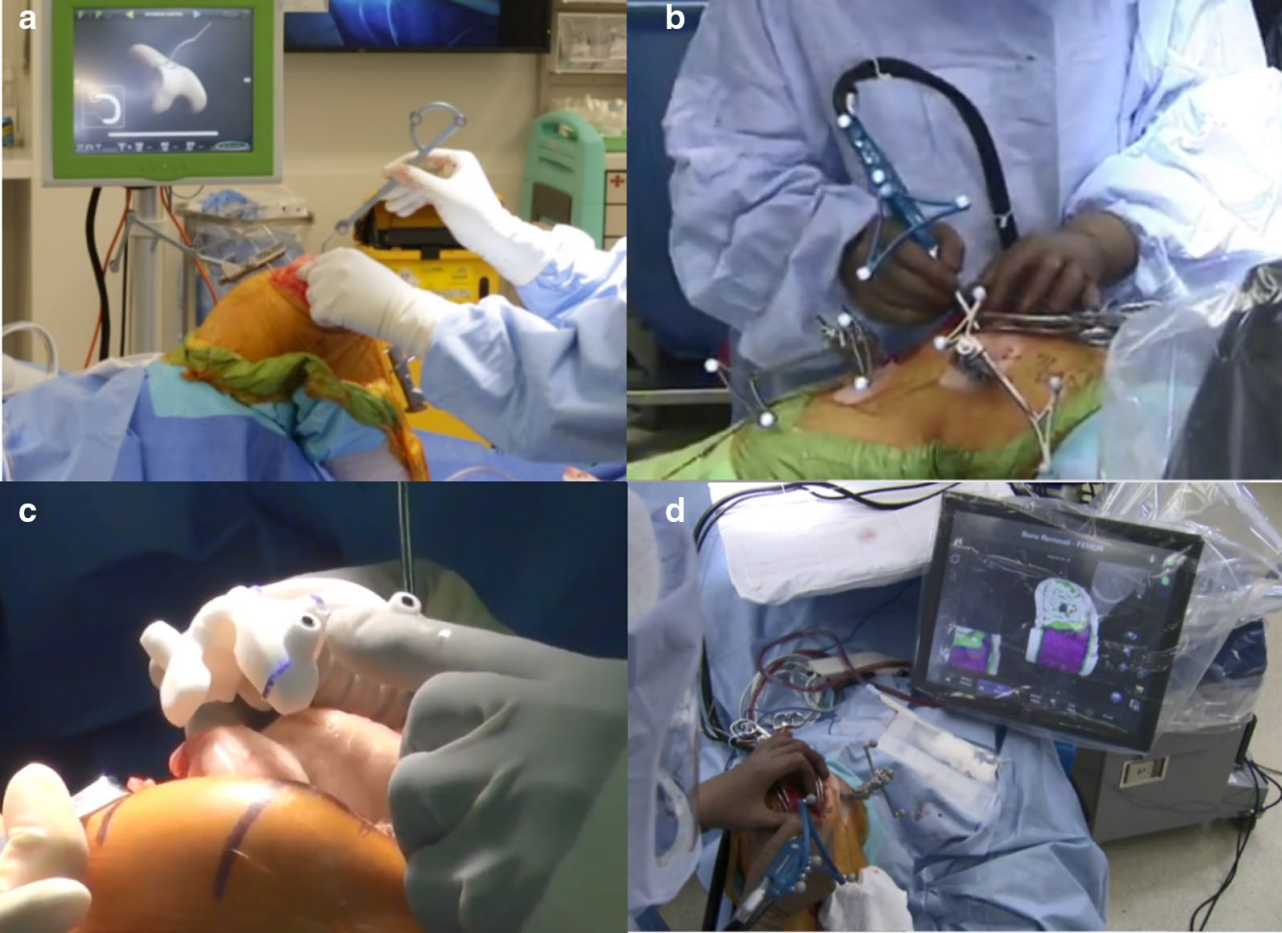
**a** Computer Navigation,** b**, **d** Robotic-assisted UKA, **c** Patient-specific instrumentation (PSI)

As any industry evolves through different phases in due course of its development [174, 175]. UKA industry, in search of an avenue for excellent outcomes and the persistent concern of its long-term survival, has integrated computer assisted technologies. These are the following: (a) computer navigation UKA, (b) patient-specific instrumentation (PSI) and implant with the help of three-dimensional (3D) printing, and (c) robotic-arm-assisted UKA (Fig. 5).

Real-time data are provided by the integration of computer technology, helping surgeons to minimize errors, especially with minimally invasive surgery. Overcorrection of limb alignment can be avoided, preventing osteoarthritis in the uninvolved compartment, thus delaying reoperation. The posterior condylar offset ratio is superior than with a conventional jig-based technique. Also, the chances of injury to essential soft tissue, especially MCL, are fewer with these newer modalities. There is higher proposed accuracy in achieving planned femoral and tibial cuts, limb alignment, soft-tissue and ligament balancing, implant position, and restoration of the native joint line. Thus, these computer assisted techniques are expected to help in the execution of surgical goals in the operating room for an even less experienced surgeon. As this eliminates the significant learning curve, inexperienced surgeons may replicate the clinical outcomes of superior function and higher longevity with UKA. Besides, the proponents of advanced technology claim improved pain control, decreased opioid analgesia dosage, and better in-patient physiotherapy than conventional UKA.

Konyves et al. revealed better implant positioning in computer-assisted navigated UKA and no difference in survivorship at 9 years compared with a conventional manual technique [176]. In a prospective study by Perlick et al., two groups of 20 UKA replacements each were operated either using a computed tomography (CT)-free navigation system or the conventional minimal invasive technique [177]. The results revealed a significant difference between the two groups in favor of navigation. In the computer-assisted group, 95% of UKAs were in a range of 4–0 degree varus (mechanical axis) compared with 70% in the conventional group. They concluded that the chances of overcorrection is diminished by real-time information about the leg axis at each step during the operation. Saragaglia et al. compared computer navigation and conventional surgery for revision of UKA to TKA [178]. They found no significant difference in the radiological target of a postoperative HKA angle at least in the hands of an experienced surgeon. However, they suggested that this technique could provide precious assistance to less experienced surgeons performing this surgery.

A prospective study of 27 patients by Cobb et al. comparing conventional jig-based UKA versus robotic UKA found that all patients undergoing robotic UKA had femorotibial axis in the coronal plane within 2° of the planned position compared with only 40% in those undergoing conventional jig-based UKA [179]. Similarly, using postoperative CT scans in 62 robotic UKAs versus 58 conventional UKAs, Bell et al. showed that robotic UKA reduced root mean square errors in achieving planned femoral and tibial implant positioning [180]. Herry et al., in their retrospective study reviewing plain radiographs of UKAs, found improved restitution of the native joint line with robotic-guided surgery compared with conventional jig-based UKAs [181].

Van den Heever et al. showed lower contact stresses and more uniform stress distribution when using patient-specific instruments and implants [182]. Jaffry et al. investigated implant positioning among PSI UKA, conventional-instrumented UKA, and robotic-assisted UKA; their results showed that PSI UKA provided more accurate positioning than conventional instrumentation and no difference between robotic-assisted and PSI UKA, and finally concluded that PSI UKA took half the time of robotic-assisted UKA to the implant [183]. However, Ollivier et al. concluded that claiming PSI improves alignment, pain, or function cannot justify the extra cost and uncertainty related to this technique [184]. A similar conclusion was reached by Kerens et al. comparing the radiographic positioning of implants in 30 conventional Oxford UKA with 30 patient-specific guided UKA. They found no statistically significant differences between the two groups [185].

Every new technology comes with its own set of disadvantages. There is an extra cost involved for the new high-end devices such as robotics, 3D printing, and computer navigation instruments [186]. Besides, there is an added cost of training surgery staff for these devices and instruments. Moreover, in some advanced technology, we require a preoperative CT scan for surgical planning which, apart from increasing the cost of surgery, also risks the patient’s health by exposing him/her to radiation hazard. There is also a risk with the newer automated technology that the very learning curve which assistive technology seeks to circumnavigate might simply be transferred from the operating theater to the computer planning stage. In the initial stages of starting navigated or robotic-assisted UKA, there can be increased time durations for surgery, which can be frustrating for an experienced surgeon who is used to quick procedures. Additionally, the already cramped valuable space of theater gets filled up with extra logistics, instruments, and equipment. Furthermore, there is also a need to prevent surgeons from becoming technicians who are unable to independently identify and address unexpected errors in the process. Nonetheless, long-term follow-up longitudinal studies and well-planned RCTs to allow the widespread use of these new assistive technologies are still lacking.

## Conclusions

The superior functional outcomes, cost-effectiveness, and improved survivorship have led to the resurgence of UKA in the past decade. Surgeons should consider UKA as a part of their armamentarium for treating knee osteoarthritis. Surgeons should aspire for optimal UKA usage in carefully selected patients along with optimal surgical technique to increase the survivability of UKA prostheses and achieve the true and full potential of UKA.

## Data Availability

Yes.
